# In silico study of bioactive compounds derived from Indonesian marine invertebrates as a novel antituberculosis agent

**DOI:** 10.55730/1300-0144.5923

**Published:** 2024-09-20

**Authors:** Ayu MASYITA, Eris SEPTIANA, Asep BAYU, Bustanussalam BUSTANUSSALAM, Jonathan A. PANGGABEAN, Firdayani FIRDAYANI, Tutik MURNIASIH

**Affiliations:** 1 Research Center for Vaccine and Drugs, Research Organization for Health, National Research and Innovation Agency (BRIN), Cibinong, Bogor, Indonesia; 2 Department of Pharmaceutical Sciences, Faculty of Pharmacy, Hasanuddin University, Makassar, Indonesia; 3 PT Biotek Rekayasa Indonesia, Jakarta Timur, Indonesia

**Keywords:** Marine natural products, molecular docking, antituberculosis

## Abstract

**Background/aim:**

Tuberculosis (TB) has become the world’s deadliest disease. The lack of an effective therapeutic drug to treat it is one of the obstacle for doctors. Today, multidrug-resistant TB cases are increasing. Investigating these new drug should be given intensive and careful consideration. Marine invertebrates are valuable since they produce a large number of active compounds, and screening of these active compounds is very important.

**Materials and methods:**

Anti-TB screening of compounds derived from marine invertebrates was performed via the in silico method. Three-dimensional structures of pantothenate kinase (MtPanK type 1, PDB ID: 4BFT), *Mycobacterium tuberculosis* InhA (PDB ID: 2X23), protein kinase B (PDB ID: 5U94), and β-ketoacyl acyl carrier protein synthase I (MtKasA, PDB ID: 2WGE) were used as the protein targeted receptors.

**Results:**

The molecular docking analysis showed that the potential candidate compounds with the lowest docking score were 19-hydroxypsammaplysin Q, 19-hydroxypsammaplysin S, psammaplysin L, and psammaplysin K dimethoxy acetal. Several compounds, such as molamide C and the manzamine group, are also potential anti-TB compounds.

**Conclusion:**

This study showed that psammaplysin groups have potential as anti-TB compounds. Further laboratory experiments should be done to confirm the in silico data.

## 1. Introduction

Tuberculosis (TB) has been a global health concern for many years, particularly in developing countries [1]. Recently, the World Health Organization reported that it causes 1.6 million deaths annually, and is a major cause of mortality and morbidity worldwide [2]. This has been made worse due to the rising prevalence of extensively drug-resistant TB (XDRTB) and multidrug-resistant TB (MDRTB) against *Mycobacterium tuberculosis* (Mtb) infection [3,4]. In addition, TB is more complex because of the distinct cell envelope resulting from Mtb [5,6]. Therefore, the search for novel chemical entities to combat this disease and new regimens that will reduce the duration of TB therapy is ongoing [7].

Indonesia, with its 17,500 islands and 81,000 km of coastline, is the largest archipelagic country in the world and is recognized as a megabiodiversity of marine species. Like all other living things, marine organisms produce a variety of metabolites, both primary and secondary, with distinct chemical characteristics, as a result of living in harsh environments. Despite their role as a chemical defense in marine organisms, these metabolites have been shown to exhibit a variety of biological properties that are crucial in drug research and discovery [8,9].

Between 1970 and 2017, about 732 compounds were isolated from Indonesian marine species. Generally, most of them have shown cytotoxic (16.7%) and antibacterial activity (5.9%) [10]. To the best of our knowledge, there is still limited comprehensive data on the potential of Indonesian marine species as natural anti-TB agents.

Molecular docking has emerged as a valuable approach for contemporary drug discovery, enabling the prediction of the binding affinities between small molecules and the intended therapeutic targets [11]. In this study, this approach was utilized for the virtual screening of compounds derived from Indonesian marine resources and to identify potential lead compounds for anti-TB activity. Some proteins from Mtb have been examined as therapeutic targets, such as pantothenate kinase (PanK) [12], InhA enzyme [13], protein kinase B (PknB) [14], and β-ketoacyl acyl carrier protein synthase I (MtKasA) [15].

## 2. Materials and methods

### 2.1. Preparation of the protein targets

Four protein targets were selected as potential receptors based on the available literature and experimental evidence implicated in Mtb infection. Three-dimensional (3D) structures of Mtb PanK (MtPanK type 1, PDB ID: 4BFT), *M. tuberculosis* InhA (PDB ID: 2X23), PknB (PDB ID: 5U94), and MtKasA (PDB ID: 2WGE) were retrieved from the Protein Data Bank (www.rcsb.org/pdb) in .pdb format. The resolution, complex with the ligand, residues of the active site, and source organisms were taken into consideration when choosing the crystal structures.

### 2.2. Preparation of the ligands

About 628 two-dimensional conformers derived from marine invertebrate (including tunicates, soft coral) and marine algae and soft corals were converted to 3D conformers using ChemOffice software package (PerkinElmer Inc., Waltham, MA, USA), subjected to energy minimization, saved in .mol2 format, and then utilized for the modeling study. The positive controls were chosen based on a literature survey for comparison.

### 2.3. Molecular docking

Molecular docking was performed using Molegro Virtual Docker 6.0 [16]. The workspace was cleared of all water molecules that were near the receptors. The best docking scores (Rerank and hydrogen bond scores) were taken into consideration for further examination since they had the lowest binding free energies (expressed as negative values). The docking results were visualized and analyzed using Discovery Studio Visualizer (BIOVIA, San Diego, CA, USA).

### 2.4. Drug likeness and absorption, distribution, metabolism, and excretion (ADME) profiles

Drug likeness was determined using Lipinski’s rule of 5 (RO5). Important ADME profiles along with properties under RO5 such as H-bond donors (HBD) less than 5, hydrogen bond acceptors (HBA) less than 10, Moriguchi Log P (M Log P) less than 4.15 or calculated Log P (C Log P) less than 5, and molecular weight (MW) less than 500 Da. Other physicochemical properties, such as the topological polar surface area (TPSA, less than 140 Å^2^), were also detected. SwissADME (http://www.swissadme.ch) was used to check these properties [17].

## 3. Results

### 3.1. Inhibition of the MtPanK

MtPanK is an attractive drug target in Mtb [18]. Using adenosine triphosphate ATP as a phosphate donor, pantothenate (vitamin B5) is transformed into 4′-phosphopantothenate in the first and rate-limiting step of the coenzyme A (CoA) biosynthesis pathway, which is catalyzed by MtPanK. Because CoA is an essential cofactor for the regulation of important metabolic enzymes in many physiological pathways, including the production and catabolism of lipids, this pathway is highly appealing as a source of novel therapeutic targets [19]. Additionally, the gene CoaA, which codes for the type I PanK, is present in the Mtb genome as a single copy [20]. In Mtb, transposon mutagenesis and gene knockout experiments have demonstrated the crucial role of CoaA. Therefore, there may be Mtb CoaA inhibitors that are bacterial-specific and barely interact with the human enzyme [21].

**Figure 1:**
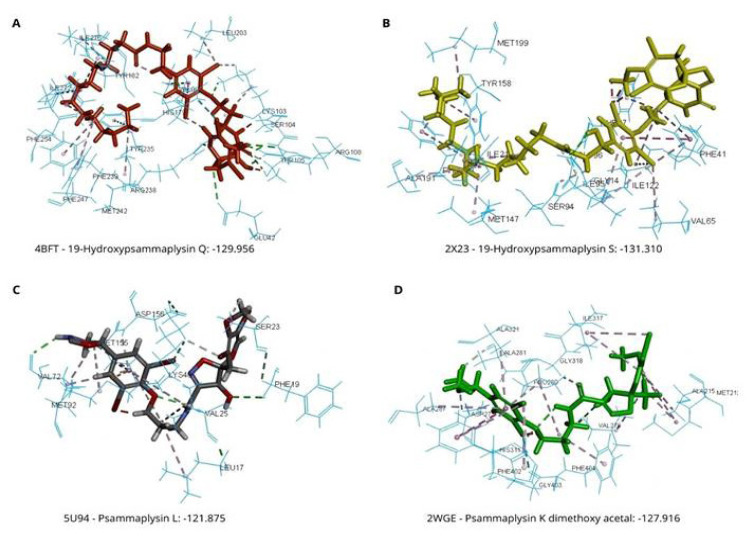
Docking pose of the top selected compounds with protein targets.

In this study, the 19-hydroxypsammaplysin Q isolated from *Acanthostrongylophora strongylata* [22] showed the lowest docking score (−129.956) and was optimally positioned in the active site of the MtPanK enzyme (Table 1) (Figure 1A). Figure 1A shows the interaction of 19-hydroxypsammaplysin Q with the binding site of the MtPanK (PDB 1D: 4BFT). Four hydrogen bonds formed between the spirooxepinisoxazoline moiety (subunit A) with GLU42, THR105, ARG108, and ARG238. Furthermore, the moloka’iamine moiety (subunit B) formed two hydrogen bonds with LYS103 and HIS179. In addition, pi-alkyl interactions formed with amino acid residues TYR182, TYR235, PHE239, MET242, PHE247, PHE254, ILE272, and ILE276.

### 3.2. Inhibition of the cell wall mycolic acid synthesis

The main components of the mycobacterial cell wall are known as mycolic acids, which assist bacteria in evading the host immune system and shield them from antibiotic resistance [23]. Therefore, it is possible to investigate the enzymes that are responsible for controlling and biosynthesizing mycolic acids as possible therapeutic targets to eradicate Mtb [24]. Enoyl acyl carrier protein reductase (InhA), a member of the NADH-dependent acyl carrier protein reductase family, is an essential enzyme involved in the synthesis of fatty acids, primarily mycolic acid. The clinical success of isoniazid as an InhA inhibitor indicates that InhA is a well-validated target for Mtb [25].

Similar to the docking results herein for MtPanK inhibitors, it was also found that the psammaplysin derivative, 19-hydroxypsammaplysin S, isolated from *A. strongylata*, showed the strongest activity against Mtb InhA (PDB 1D: 2X23) with a molecular docking score of −131.31. This score was the lowest when compared to commercial drugs, especially with isoniazid (Table 2). The current study revealed that the amine and carbonyl oxygen component in the moloka’iamine moiety formed two hydrogen bonds with GLY14 and GLY99, respectively. Furthermore, pi-alkyl interactions were formed with amino acid residues VAL65, PHE97, and ILE122. Moreover, pi-pi stacking was encountered between the 1moloka’iamine ring with PHE41 (Figure 1B).

### 3.3. Inhibition of the Mtb PknB (MtPknB)

MtPknB is one of the serine/threonine Pkns (Ser/Thr PknB), which regulate Mtb’s phosphorylation-mediated signal and act in a manner similar to eukaryotic kinases [26]. MtPknB is involved in many mycobacterial cellular functions, including cell division, cell wall synthesis, cell metabolism, etc. [27]. In addition, it is necessary for the cells to reactivate following a hypoxic state [28]. Therefore, Ser/Thr PknB is essential to maintain mycobacterial growth, which lends credence to the concept that Pkn inhibitors could be developed as novel, potentially effective anti-TB medications [29].

The molecular docking revealed that psammaplysin L isolated from *A. strongylata* showed the lowest docking score against MtPknB (PDB 1D: 5U94) (Table 3). Moreover, it was more potent compared to commercial drugs like isoniazid, rifampicin, pyrazinamide, ethambutol, and streptomycin (STR), for which the docking scores range from −52.527 to −80.798. Two hydrogen bonds formed between the spirooxepinisoxazoline ring with PHE9 and SER23 (Figure 1C). Furthermore, the moloka’iamine ring formed hydrogen bonds with VAL72 and ASP156. The bromo atom in the spirooxepinisoxazoline and moloka’iamine ring also generated a hydrogen bond with LYS40. Additionally, van der Waals interactions formed with amino acid residues GLY18, GLY20. GLU24, ALA38, GLU59, ALA73, GLU93, MET145, and VAL154.

### 3.4. Inhibition MtKasA

MtKasA is an important protein target in the biosynthesis of the mycobacterial cell wall [15]. One distinctive characteristic of the mycobacterial cell wall is the presence of mycolic acids [30]. These extremely long-chain fatty acids are created by a variety of fatty acid synthases (FAS), and their production has been connected to the capacity of mycobacteria to endure in the host and withstand numerous antibiotics. The mycobacterial type II FAS pathway, which is unique to bacteria, contains MtKasA [31]. According to recent research, MtKasA has been reported as a promising target for Mtb [32].

The most potential anti-Tb compound based on the molecular docking with the target MtKasA (PDB 1D: 2WGE) was psammaplysin K dimethoxy acetal, with a docking score of −127.916 (Table 4). Among the antibiotics, STR showed the lowest molecular docking score (−114.119), but this value was more than that of the psammaplysin K dimethoxy acetal (Table 5). H-bonding was observed between the carbonyl oxygen component in the spirooxepinisoxazoline moiety with VAL278 and GLY318. This was in addition to the hydrogen bond that existed between the hydroxyl group and the brom atom in the moloka’iamine moiety with ASP273 and HIS311, respectively (Figure 1D).

### 3.5. Drug likeness

Drug-likeness properties were evaluated to establish whether a compound satisfied the requirements of the criteria as a drug. In terms of Lipinski’s RO5, the compounds should be in line with these criteria: 1) the Lipinski rule with at most one violation (MW ≤500 Da, logP ≤5, hydrogen acceptor count ≤10, and hydrogen donor count ≤5); 2) TPSA ≤ 75 Å2; 3) human intestinal absorption ≤0.7; 4) human oral bioavailability 30% ≤0.7; 5) the drug’s half-life ≤0.8; 6) carcinogenicity ≤0.3; and 7) Ames test toxicity ≤0.3. [33]. In this study, psammaplysin L and psammaplysin K dimethoxy acetal were more qualified for drug-likeness than 19-hydroxypsammaplysin Q and S, especially for the cLogP data requirement. Nevertheless, the TPSA value of all these potential compounds was higher than the required value, meaning that the membrane cell permeability was poor. Further optimization and wet laboratory experiment confirmation was needed to obtain the best drug lead compounds.

Meanwhile, the capacity of a compound to go through the ADME processes is typically indicated by the evaluation of its ADME profiles. The blood-brain barrier (BBB), permeability glycoprotein (Pgp) substrate, and gastrointestinal (GI) absorption data were used as typical features. It was revealed that 19-hydroxypsammaplysin Q, 19-hydroxypsammaplysin S, and psammaplysin L had similar ADME properties, such as low-level absorption in the GI tract (Table 6). This indicated that they would not be able to transport across the BBB. In contrast, psammaplysin K dimethoxy acetal showed a high level of GI absorption, which was probably because of its lowest MW compared to the other compounds. Additionally, none of the compounds were able to cross the barrier, suggesting that they might not be harmful to the brain.

## 4. Discussion

The results of the molecular docking to the marine metabolites against the targeted protein representing the anti-TB mechanism showed several groups of compounds that should be considered for further analysis.

### 4.1. Psammaplysins group

The psammaplysin groups were observed to be markers of the Verongiidae sponges group. About 42 psammaplysin compounds were successfully isolated from the marine sponges, mostly from 10 species of the family Verongiidae [34]. The biosynthetic pathway of psammaplysins is the same with that of the bromotyrosin group via benzene oxide-oxepin intermediates. The structure of psammaplysin consists of two dibrominated moieties, 8,10-dibromo-4-hydroxy-9-methoxy-1,6-dioxa-2-azaspiro[4.6]undeca-2, 7, 9-triene-3-carboxylic acid (subunit A), and 3-(4-(2-aminoethyl)-2, 6-dibromophenoxy)propan-1-amine subunit (subunit B, moloka’iamine), linked together through an amidic linkage between the carboxylic moiety (C-9) of the substituted spirooxepinisoxazoline unit and the terminal amino group at C-10 of the moloka’iamine [34]. The evaluation of their biological activity reported that psammaplysins A and B inhibited Mtb detoxification enzyme mycothiol-S-conjugate amidase in a fluorescence-detected assay [35].

The spirooxepinisoxazoline ring system is an essential element for the activity of psammaplysins. It is indicated by the increasing activity of the N-terminal substitution with a cyclopentene-dione moiety, e.g., in psammaplsyins E and 19-hydroxypsammaplysin E. Or the 4-chloro-2-methylenecyclopentane-1,3-dione moiety, e.g. in psammaplysin X and 19- hydroxypsammaplysin X. The N methyl group, shown in psammaplysin F, or a urea moiety, as in psammaplysin Z, was reported to diminish the activity. While the 19-OH group, as in psammaplysin B versus psammaplysin A was lost in the activity. On the contrary, psammaplysin D lacked activity (GI50 > 10 μM), possibly due to high lipophilicity. The N-methyl in psammaplysin F and urea moiety in psammaplysin Z were reported as the important functional group that contribute to the activity [34].

**Figure 2:**
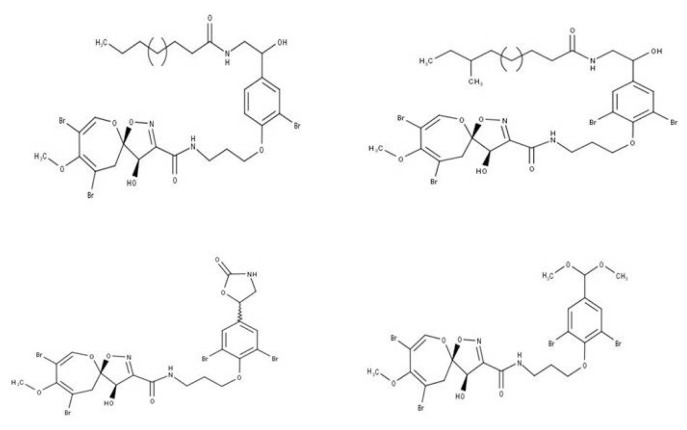
Diversity structure of the potential anti-TB from the psammaplysins group.

However, the potential anti-TB compounds, based on this molecular docking analysis were 19-hydroxypsammaplysin Q, 19-hydroxypsammaplysin S, psammaplysin L, and psammaplysin K dimethoxy acetal (Figure 2). Psammaplysin L was previously reported as antimalarial, while 19-hydroxypsammaplysin Q, 19-hydroxypsammaplysin S, and psammaplysin K dimethoxy acetal were inactive [22]. The experimental bioactivity data of 19-hydroxypsammaplysin Q, 19-hydroxypsammaplysin S, and psammaplysin K dimethoxy acetal are not currently available.

### 4.2. Mollamide C

The in silico studies to find a new anti-TB agent using MtPanK inhibition showed that mollamide C was a potential anti-TB compound, with a docking score of −127.563. This computational study gives a significant result to the experimental data that is found by [36]. Mollamide C is a hexapeptide cyclic isolated from the Indonesian tunicate *Didemnum molle* and has been investigated as an anti-Mtb compound [36]. A study investigating cyclic peptide compounds reported that the cyclic conformation of peptides increases their stability, resulting in good absorption, distribution, and metabolic excretion, making them suitable for pharmaceutical applications [37].

Previous research reported cyclohexadepsispeptideswere isolated from the family Enniatins which showed remarkable antibiotic and cytotoxic activity [38–41]. In a detailed analysis of their mechanism of action in the transmembrane cation translocation, the formation of the transmembrane pore of the cyclic peptide was affected by antibacterial activity through cell lysis and leakage of the intracellular content [37]. The interaction between positively charged cyclic peptides with the negatively charged bacterial lipid head group affected the strength of their antibacterial activity [42–44]. Their antimicrobial activity was dependent on the size of the oligomer and the cationic side chain of the cyclic peptide. The thiazole side group in mollamide C played an important role in its bioactivity [36].

### 4.3. Manzamines group

Acanthomanzamine C is a manzamine alkaloid containing one macrocyclic ring. Manadomanzamines A and B were reported from the Indonesian sponge, *Acanthostrongylophora* sp. [45]. These compounds exhibited tubercular effects against Mtb, with minimum inhibitory concentration (MIC) values of 1.9 and 1.5 μg/mL, respectively. Rifampicin was used as a control and showed a tubercular effect, with MIC values of 0.16 μg/mL [46].

The presence and position of the hydroxyl group on the β-carboline moiety may play an important role in the biological activity of these analogs, as seen from comparing manzamine B 2 and its hydroxy derivatives manzamine C and zamamidine B (MIC: 6.74, 0.69, and 52.07 μM). Due to their unique and diverse structures, the manzamine alkaloids have been attractive targets for investigation, including pharmacological, biogenetic, and synthetic studies [47–54]. In the biosynthetic pathway, DielsAlder-type reactions are suggested to be efficient mechanisms for constructing the pivotal hydroquinoline central core [47,48,51,54,55]. Knowledge developed from these biosynthetic investigations has stimulated the use of new chemical reactions for manzamine formulation and may improve the efficiency of their synthesis [51].

Herein, the most potential anti-TB compounds with the lowest docking scores were 19-hydroxypsammaplysin Q, 19-hydroxypsammaplysin S, psammaplysin L, and psammaplysin K dimethoxy acetal. Of these, only psammaplysin L was previously shown to have activity against *P. falciparum*. The current study is the first to report the potential of the psammaplysin group as anti-TB compounds using virtual screening. Further experimental studies should be performed to confirm the antimycobacterial activity of these compounds.

## Figures and Tables

**Table 1:** The top 13 docking scores of marine bioactive compounds interact with pantothenate kinase (PDB 1D: 4BFT).

Bioactive compound	Source	Docking score
19-Hydroxypsammaplysin Q	*Acanthostrongylophora strongylata*	−129.956
Mollamide C	*Didemnum molle*	−127.563
Psammaplysin W	*A. strongylata*	−124.921
Acanthomanzamine C	*A. ingens*	−121.647
19-Hydroxypsammaplysin T	*A. strongylata*	−120.671
Pre-neo-kauluamine	*A. ingens*	−120.283
Psammaplysin L	*A. strongylata*	−119.083
Melophluoside B	*Melophlus sarasinorum*	−117.262
19-Hydroxypsammaplysin T	*A. strongylata*	−117.203
Psammaplysin S	*A. strongylata*	−116.32
Psammaplysin K dimethoxy acetal	*A. strongylata*	−112.553
Nakijiquinone V	*Dactylospongia elegans*	−112.466
(−)-leptoclinidamine B	*Leptoclinides dubius*	−109.864

**Table 2:** The docking scores of bioactive compounds derived from Indonesian marine invertebrates with targeted Mtb InhA (PDB 1D: 2X23).

Bioactive compound	Source	Docking score
19-Hydroxypsammaplysin S	*A. strongylata*	−131.310
Cladielloide A	*Cladiella sp*.	−122.897
Psammaplysin L	*A. strongylata*	−118.675
(−)-leptoclinidamine B	*L. dubius*	−118.161
Spironaamidine	*Leucetta microraphis*	−109.518
Nakijiquinone V	*D. elegans*	−108.050
Ingenine D	*A. ingens*	−106.558
Acanthocyclamine A	*A. ingens*	−106.246
3,4-epoxy-nephthenol acetate	*Nephthea sp*.	−105.415
Chloroscabrolide A	*Sinularia sp*.	−105.375
Sarcofuranocembrenolide B	*Sarcophyton sp*.	−105.008
Manadoperoxide D	*Plakortis cfr. simplex*	−104.641
Psammaplysin K	*A.strongylata*	−103.932
Halioxepine	*Haliclona sp*.	−103.019
Psammaplysin T	*A. strongylata*	−102.954
Psammaplysin K dimethoxy acetal	*A. strongylata*	−102.682

**Table 3:** The docking scores of bioactive compounds derived from Indonesian marine invertebrates with protein kinase B (PDB 1D: 5U94).

Bioactive compound	Source	Docking score
Psammaplysin L	*A. strongylata*	−121.875
Lissoclibadin 4	*Lissoclinum cf. badium*	−119.520
Haloirciniamide A	*Ircinia sp*.	−114.879
Ingenine D	*A. ingens*	−113.985
(−)-leptoclinidamine B	*L. dubius*	−113.192
Psammaplysin M	*A. strongylata*	−113.091
Psammaplysin K dimethoxy acetal	*A. strongylata*	−110.129
Nakijiquinone V	*D. elegans*	−109.908
Celebeside A	*Siliquariaspongia mirabilis*	−109.312
19-Hydroxypsammaplysin E	*A. strongylata*	−109.127
Seribunamide A	*Ircinia sp*.	−108.588
Sagitol C	*Oceanapia sp*.	−107.110
Psammaplysin N	*A. strongylata*	−106.799

**Table 4:** The docking scores of bioactive compounds derived from Indonesian marine invertebrates with MtKasA (PDB 1D: 2WGE).

Bioactive compound	Source	Docking score
Dispacamide E	*Stylissa massa*	−125.947
3-debromolatonduine B methyl ester	*Stylissa sp*.	−117.690
Psammaplysin S	*A. strongylata*	−112.947
Acantholactam	*A. ingens*	−110.864
(−)-leptoclinidamine B	*L. dubius*	−110.601
Ingenine D	*A. ingens*	−109.818
Ingenine C	*A. ingens*	−108.737
Variabine B	*Luffariella variabilis*	−105.765
Cortistatin F	*Corticium complex*	−105.027
Psammaplysin Q	*A. strongylata*	−103.283
Leptoclinidamide	*L. dubius*	−102.456
19-Hydroxypsammaplysin U	*A. strongylata*	−101.920
Psammaplysin Q	*A. strongylata*	−103.283
Leptoclinidamide	*L. dubius*	−102.456
19-Hydroxypsammaplysin U	*A. strongylata*	−101.920

**Table 5:** The docking scores of antitubercular medications (FDA approved) with several potential receptors implicated in Mtb infection.

Ligand	Protein target			
	4BFT	2X23	5U94	2WGE
Isoniazid	−45.459	−49.894	−54.429	−68.390
Rifampicin	−113.439	−55.734	−62.022	16.213
Pyrazinamide	−43.066	−48.781	−52.527	−66.345
Ethambutol	−56.622	−74.534	−69.867	−35.916
Streptomycin	−113.116	−72.686	−80.798	−114.119

**Table 6:** Drug likeness and ADME profiles of top-selected compounds.

Compound	MW	HBA	HBD	cLogP	TPSA	GIA	BBB	P-gpS
19-hydroxy psammaplysin Q	959.406	9	4	8.30	328.409	Low	No	Yes
19-hydroxy psammaplysin S	1001.487	9	4	9.326	347.504	Low	No	Yes
Psammaplysin L	775.03	9	3	3.25	136.94	Low	No	Yes
Psammaplysin K dimethoxy acetal	764.05	9	2	3.81	117.07	High	No	Yes

MW: molecular weight, HBA: hydrogen bond acceptor, HBD: hydrogen bond donor, TPSA: topological polar surface area, BBB: blood-brain barrier, GIA: gastrointestinal absorption, P-gpS: permeability glycoprotein substrate.

## Data Availability

Data used in this study are available upon request.
